# Sleep Disorders: Pathogenesis and Therapeutic Interventions

**DOI:** 10.1002/mco2.70130

**Published:** 2025-03-10

**Authors:** Cheng Liu, Zhigang He, Yanqiong Wu, Yanbo Liu, Zhixiao Li, Yifan Jia, Hongbing Xiang

**Affiliations:** ^1^ Department of Anesthesiology, Hubei Key Laboratory of Geriatric Anesthesia and Perioperative Brain Health, Wuhan Clinical Research Center for Geriatric Anesthesia Tongji Hospital, Tongji Medical College, Huazhong University of Science and Technology Wuhan Hubei China; ^2^ Department of Pain Renmin Hospital of Wuhan University Wuhan Hubei China; ^3^ Key Laboratory of Anesthesiology and Resuscitation (Huazhong University of Science and Technology), Ministry of Education Wuhan China

**Keywords:** brain, neural circuit, neuronal plasticity, neurotransmitters, sleep disorder

## Abstract

Sleep disorder significantly disrupts the quality of life for patients. Although it is clinically acknowledged, the fundamental neuropathological mechanisms are still not understood. Recent preclinical research has been directed toward understanding the fundamental mechanisms underlying the sleep deprivation and sleep/wake dysregulation. Sleep disorder is linked to changes in the structure and function of the neural basis of cognition. We reviewed the neural circuits related to sleep disorders, along with alterations in connectivity and brain region functions, based on advancements in electrophysiology and optogenetic/chemogenetic techniques. We subsequently outline the cellular and molecular modifications linked to sleep disorders in preclinical studies, primarily involving changes in neuronal metabolism, electrophysiological activity, synaptic plasticity, and glial cells. Correspondingly, on the basis of the crosstalk between the brain and peripheral organs, we elucidate the underlying mechanisms of the involvement of celiac disease and hepatic disease in the pathogenesis of sleep disorders. In this review, we mainly discussed the pathogenesis at molecular, cellular, and neural circuit levels that contribute to sleep disorder. The review also covered potential strategies for treating sleep disorders and future research avenues.

## Graphical abstract

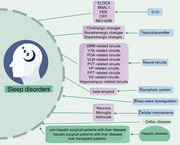

## 1 Introduction

Sleep is an innate behavior and a multidimensional process [1]. It is a critical period in the daily routine for restoring physiological and brain functions and promoting cognitive health and good mental. Sleep deprivation is a primary cause of many harmful diseases related to the brain, heart, high blood pressure, psychological changes, diabetes, and so forth. However, sleep disorders are a serious public health event globally, with rising prevalence rates and approximately 30% of the world's population is currently sleeping poorly or insufficiently [2, 3]. Although the International Classification of Sleep Disorders, 3rd edition, categorizes sleep disorders into seven major groups: insomnia, sleep‐related breathing disorders, circadian rhythm sleep–wake disorders, parasomnias, central disorders of hypersomnolence, sleep‐related movement disorders, and other sleep disorders, sleep disorders remain largely unknown. The diagnosis of these conditions is typically based on clinical symptoms and polysomnography [4, 5, 6].

Among these, insomnia, as the most prevalent form of sleep disorder, can affect 10%–15% of the general population [7, 8]. Existing literature often uses the term “sleep–wake disorders” to refer to pathological conditions where the body's internal biological clock cannot function properly or synchronize with the external day–night cycle [9, 10]. Under normal physiological conditions, the body relies on its inherent circadian rhythm to regulate daily behaviors and physiological functions. This rhythm cycles every 24 h, precisely controlling physiological processes such as sleep patterns, eating habits, body temperature fluctuations, and hormone secretion [11].

Significantly, sleep disorders might be an early indicator of mental and brain health problems, linked to cognitive and emotional disorders, as well as neurodegenerative diseases overall [12]. Patients with depression and sleep disorders frequently experience a decline in cognitive abilities linked to executive functioning, such as alertness and processing speed. Good sleep is associated with subjective perceptions of restorative sleep, memory consolidation, and reduced neurohumoral stress responses [13]. For certain patients, chronic exposure to stress is an etiologic risk factor for depression and neuroinflammation. Inflammation is not unique to depression and is a disease risk factor shared between many psychiatric disorders such as sleep disorders and metabolic disorders. The mechanism probably involves cytokines sensitizing the hypothalamic‐pituitary‐adrenal (HPA) axis, leading to a disruption in the negative feedback loop and intensifying the inflammatory response. Central inflammation, or neuroinflammation, is worsened by peripheral inflammation through mechanisms such as blood–brain barrier disruption, immune cell movement, and glial cell activation. Upon activation, glial cells discharge cytokines, chemokines, and reactive oxygen and nitrogen species into the extrasynaptic region, leading to dysregulation of neurotransmitter systems, imbalance of excitation to inhibition ratios, and disruption of neural circuit plasticity and adaptation [14].

Also, diet is thought to influence sleep characteristics [15]. Known for its substantial impact on human health, the Mediterranean diet is considered an important dietary pattern. It has been the subject of extensive studies for its health benefits and environmental sustainability, while at the same time it is a palatable diet that is also relatively accessible to non‐Mediterranean cultural populations. There is significant proof that sticking to the Mediterranean diet can decrease the likelihood of developing cardiometabolic disorders, neurodegenerative diseases, and specific cancers. Primarily plant‐based, the Mediterranean diet is abundant in antioxidant vitamins such as C and D, along with phytochemicals, which may help to prevent microglial cell activation, reduce the production of proinflammatory cytokines, and ultimately suppress neuroinflammation [16].

**FIGURE 1:**
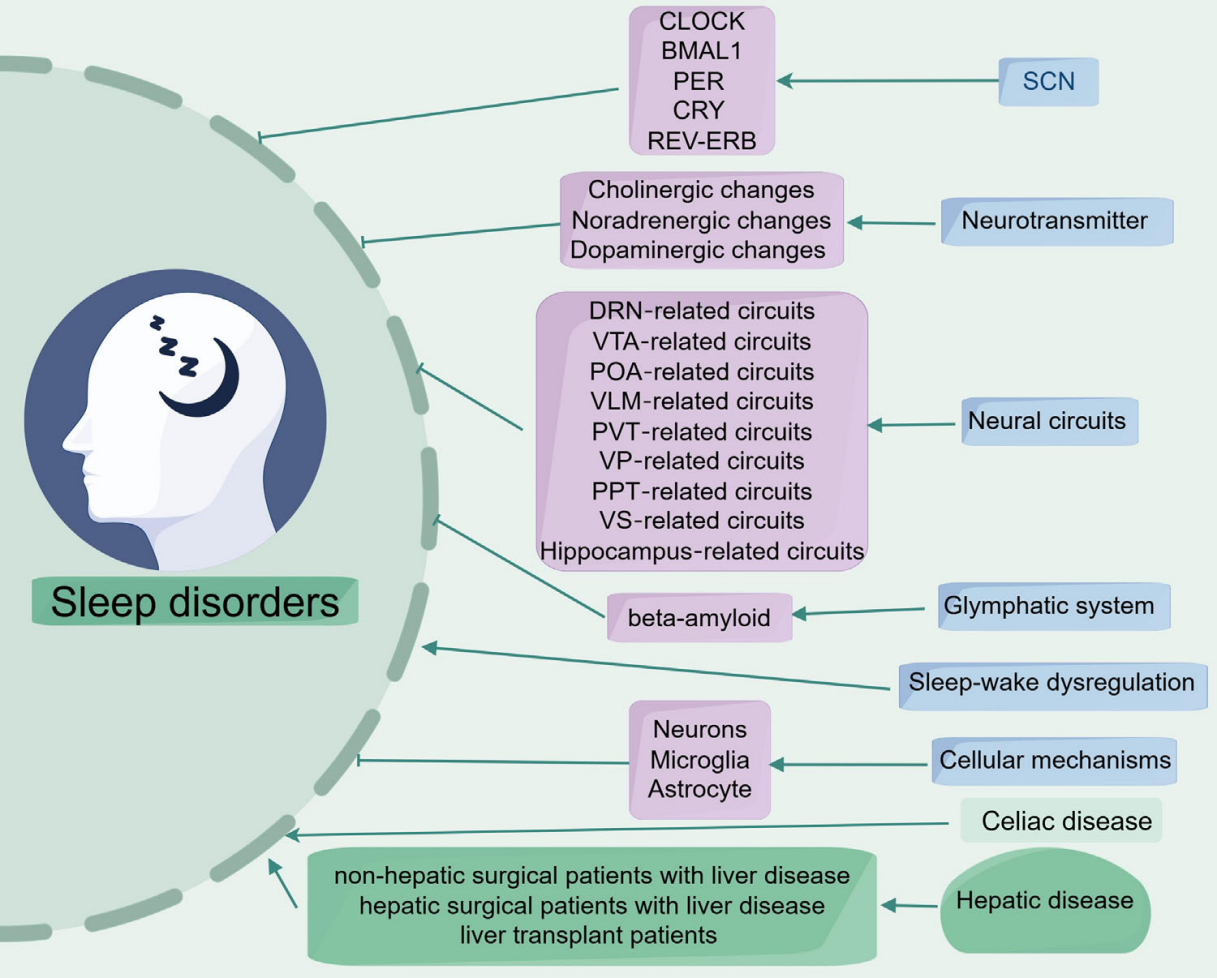
The cellular and molecular alternations related to sleep disorder and the underlying mechanisms of the involvement of peripheral organ disease in the pathogenesis of sleep disorders. DRN, dorsal raphe nucleus; POA, preoptic area; PPT, pedunculopontine tegmentum; PVT, paraventricular nucleus; SCN, suprachiasmatic nuclei; VLM, ventrolateral medulla; VP, ventral pallidum; VS, ventral striatum; VTA, ventral tegmental area.

Sleep disorders are affected by numerous factors, and the clinical manifestations and pathogenesis of different insomnia subtypes vary. In this review, we outline the neural circuits linked to sleep disorders and examine alterations in brain region connectivity and function (Figure 1). In this part, we also describe the cellular and molecular changes associated with sleep disorders in preclinical studies, which primarily include changes in neuronal metabolism and electrophysiology, synaptic plasticity, and glial cell changes. In the next section, we also elucidate the potential mechanisms by which celiac disease (CD) and hepatic disease are involved in the pathogenesis of sleep disorders and discuss potential therapeutic strategies and future research directions for sleep disorders.

## 2 Specific Types and Animal Model of Sleep Disorders

### 2.1 Major Sleep Disorders

Sleep disorders are common, with more than one‐third of adults experiencing insomnia at some point. Insomnia is usually short‐lived at first, but becomes chronic in about 40% of cases. It is diagnosed when there is dissatisfaction with sleep (sleep‐onset or sleep‐maintenance insomnia) and symptoms such as daytime drowsiness, poor concentration, and mood disorders occur for at least three nights a week for more than 3 months. Another common disorder of sleep disorders is sleep apnea, which affects 11%–23% of women and 30%–50% of men. Symptoms consist of loud snoring, choking or gasping, breathing interruptions seen by the bed partner, excessive tiredness and sleepiness, and headaches in the morning. If left untreated, it increases the risk of type 2 diabetes and cardiovascular disease such as high blood pressure. Narcolepsy is an uncommon condition marked by extreme drowsiness, sleep paralysis, and hallucinations. They include narcolepsy type 1 (with cataplexy), narcolepsy type 2 (no cataplexy), recurrent hypersomnia (such as Kleine–Levin syndrome), and idiopathic hypersomnia (with long sleep time or without long sleep time). Circadian rhythm disorders, such as sleep latency and sleep advancement disorders, affect the timing but not the duration of sleep. Delayed sleep disorders are common in adolescents and young adults, while early sleep disorders are more common in older adults. Anisomnias, including somnambulism and night terrors, usually arise from slow‐wave sleep (SWS) and involve a variety of behaviors without vivid dream recall. Restless legs syndrome (RLS) is characterized by a desire to move limbs due to uncomfortable sensations, especially at rest and at night, leading to difficulty sleeping and daytime fatigue.

### 2.2 Rapid Eye Movement Sleep Behavior Disorder

Rapid eye movement sleep behavior disorder (RBD) is an anomalous sleep disorder characterized by a lack of normal muscle tone and unusual behavior during rapid eye movement (REM) sleep. It is associated with a variety of neurological disorders, particularly a group of neurodegenerative disorders known as alpha‐synucleinopathies, including Lewy body dementia, Parkinson's disease (PD), and multiple system atrophy. Combined with clinical, neuroimaging, and sleep‐related data, these transcranial magnetic stimulation (TMS) findings suggest the presence of cortical electrical imbalances in RBD patients. In awake subjects with idiopathic RBD (iRBD) but still without symptoms of PD or dementia, changes in intracortical facilitation (ICF) and, to a lesser extent, short‐interval intracortical inhibition (SICI) may reflect an imbalance between intracortical facilitating and inhibiting microcircuits [17]. Variations in the engagement of cortical brain structures related to sleep cycle and REM stage regulation in PD/pRBD and iRBD might indicate a shared structural foundation connecting iRBD and PD, although this pattern alone may not fully account for RBD‐related characteristics [18]. Although phenotype switchers showed greater overall progression in motor, cognitive, olfactory, and some autonomic markers than nonswitchers, the only significant difference in progression between PD and Lewy body dementia phenotype switchers was in cognitive testing [19]. RBD is a significant indicator of potential neurodegenerative disease. Patients with iRBD have an estimated 90% risk of developing a neurodegenerative disease after long‐term follow‐up: 44.8% at 6 years and from 67.5% to 81% at 10 years [20].

### 2.3 Restless Legs Syndrome

While numerous studies have suggested certain structures in the central nervous system, the dopamine system, and iron metabolism are involved in RLS, the precise anatomical basis remains unidentified. Autopsy studies of RLS patients have also shown that D2 receptors are significantly reduced in the nucleus accumbens (NAs) and correlate with disease severity, while tyrosine hydroxylase (TH) is increased in the substantia nigra [21]. In addition, the midbrain limbic dopaminergic pathway, which may be involved in RLS, originates in the ventral tegmental area (VTA) and sends fibers to certain sites, such as frontal cortical areas, including the nucleus ambiguus and limbic structures. In addition, this pathway is part of the limbic system, whose afferent nerves travel from the hippocampus and amygdala to the nucleus ambiguus and ventral striatum (VS). Research has utilized structural magnetic resonance imaging (MRI) to examine subcortical gray matter in RLS patients, aiming to identify any volume or shape abnormalities. Functional magnetic resonance imaging (fMRI) postprocessing utilized the Brain Analysis Group's Comprehensive Alignment and Segmentation Tool (FIRST) software to analyze the volume and morphology of the thalamus, caudate nucleus, NAs, pallidum, brainstem, hippocampus, and bilateral amygdala. Patients with RLS exhibited notable volume reductions in the left amygdala and left pallidum, along with substantial surface morphological changes in the bilateral amygdala. Additionally, less pronounced surface alterations were observed in the hippocampus, right caudate nucleus, left pallidum, and left chiasma.

### 2.4 Animal Model of Sleep Disorders

The animal models of sleep disorders have various forms, mainly including total sleep deprivation (TSD), selective sleep deprivation (SSD) and forced locomotion technique (FLT; Table 1). The sleep deprivation model (TSD and SSD) is used to study the pathological changes of experimental animals after REM sleep deprivation [22]. The principle of FLT is to force experimental animals to move continuously inside a power device, thereby achieving the goal of sleep deprivation. The rotary cylinder and the horizontal turntable deprivation types are currently commonly used methods. The analysis from Urushihata et al. [23] indicated that the connection of cellular activity across brain regions was modified due to sleep deprivation, suggesting that a detailed examination of the whole brain is beneficial for grasping the mechanisms through which sleep disruption influences brain function.

**TABLE 1:** A list of preclinical studies showing neuronal morphologic and functional changes in the sleep deprivation states.

**Model**	**Brain region**	**Neural morphologic and functional changes**	**References**
Chronic sleep deprivation	Prelimbic cortex	Increasing the expression of Sirt6 improves cognitive impairment caused by sleep deprivation by affecting the function of glutamatergic neurons	[22]
Acute sleep deprivation	Temporal brain region	Circadian regulation of the lactate metabolic kinetics in mice using the [^1^H–^13^C]‐nuclear magnetic resonance (NMR) technique	[24]
Prolonged sleep deprivation	Hippocampus	Following extended periods of sleep deprivation, SIRT6 supports the formation of new neurons in the hippocampus by modulating energy metabolism in developing rats	[25]
Chronic sleep deprivation	Hippocampus	Following sleep deprivation, complement activation perpetuates neuroinflammation and impairs adult neurogenesis and spatial memory in the rat hippocampus	[26]
Automated cage shaking apparatus	Hippocampus	Reducing microglia activation leads to better spatial memory and adult neurogenesis in the rat hippocampus following 48 h of sleep deprivation	[27]
Sleep deprivation (SD)‐induced cognitive impairment	Hippocampus	In rats, sleep deprivation leads to spatial memory problems due to modified neuroinflammatory responses and glial cell activation in the hippocampus	[28]
Automatic cage vibrating stimulus	Dentate gyrus	Blocking adenosine A1 receptors alleviates the negative effects of sleep deprivation on adult neurogenesis and spatial reference memory	[29]
SD‐induced cognitive deficits	Hippocampus	Adenosine from astrocytes and A1 receptor activity play a role in the impairments in hippocampal synaptic plasticity and memory caused by sleep deprivation	[30]

## 3 Pathogenesis of Sleep Disorders

### 3.1 Neurobiological Mechanisms

Various neurons and neurotransmitters in the brain are crucial for sleep regulation. Here are some key nervous systems and substances: In hypothalamus, the suprachiasmatic nucleus (SCN) of the hypothalamus is the core of the biological clock, responsible for regulating circadian rhythms. It regulates melatonin secretion by receiving light signals, thereby affecting sleep and wakefulness. In brainstem, the locus coeruleus (LC) and VTA in the brainstem participate in alertness and sleep–wake cycles. The noradrenergic neurons in the brainstem are closely related to the arousal state.

Neurotransmitters have shown a regulatory effect on sleep disorders. Gamma aminobutyric acid (GABA) is a major inhibitory neurotransmitter closely related to the induction and maintenance of sleep. GABA can significantly reduce excitability in the cortex and hippocampus. Glutamate is a major excitatory neurotransmitter that promotes wakefulness and learning. Glutamate overactivity associated with insomnia may affect sleep structure. Other neurotransmitters such as norepinephrine, dopamine, serotonin, and so forth also affect sleep by regulating mood and anxiety levels.

Melatonin is secreted by the pineal gland to regulate the sleep–wake cycle. At night, its secretion increases, promoting sleep. Research has shown that the imbalance of melatonin is associated with insomnia. As a stress hormone, cortisol typically reaches its peak in the early morning. Chronic stress can lead to high levels of cortisol, which is closely related to insomnia symptoms.

### 3.2 Genetic Factors

Early twin and family studies have shown that insomnia can be attributed to genetics. In recent years, with the rapid development of gene sequencing technology, some studies have focused on insomnia and genes [31, 32], confirming that genetic factors play an important role in the occurrence and development of insomnia. Some studies have shown that there are genetic similarities between insomnia and diseases such as depression, anxiety, and cardiovascular disease. This means that certain genetic variations may be risk factors for the co‐occurrence of insomnia and other diseases [33]. Understanding the role of these genetic factors can help us better prevent and treat related diseases.

In mammals, the light–dark cycle and associated circadian rhythms play an important role in the presentation of sleep quality and sleep disorders [34]. Existing research suggests that the SCNs in the hypothalamus are the important anatomical and physiological basis for regulating this rhythm. The SCN contains about 20,000 clusters of neurons, each exhibiting spontaneous circadian rhythm oscillations. “Clock genes” form the molecular basis of these circadian oscillations, encoding proteins involved in generating the day–night rhythm mechanism [35]. The transcription factor CLOCK and BMAL1, along with inhibitory proteins such as PER1, PER2, PER3, CRY1, and CRY2, collectively form the transcriptional–translational feedback loops (TTFLs) [11, 36]. In this system, the CLOCK/BMAL1 heterodimer triggers gene transcription, including clock proteins. These clock proteins form heterodimers (PER/CRY) and accumulate in the cytoplasm. Once reaching a certain level, they are transported into the nucleus and inhibit the activity of CLOCK/BMAL1, temporarily blocking the transcription of genes including clock proteins [37, 38, 39]. This self‐inhibition constitutes the core of the TTFLs negative feedback loop. Additionally, the CLOCK/BMAL1 heterodimer can form a positive feedback regulation by triggering the transcription of Rev‐erbs and Rors genes, promoting the expression of BMAL1 [40, 41, 42]. Moreover, up to dozens of other proteins are involved in TTFLs regulation, making the entire loop regulation more refined and reliable. Epigenetic post‐translational modifications and miRNAs also play a role in TTFLs regulation [43, 44, 45].

The influence of genetic components in certain sleep disorders is more evident. Fatal familial insomnia (FFI) is an uncommon condition marked by significant sleep issues, autonomic irregularities, motor symptoms, and unusual behavior. The disease usually manifests as primary atrophy of the thalamic nuclei and inferior olivary nucleus and extends to other brain regions as the disease progresses. This is an autosomal dominant prion disorder resulting from a D178N mutation in the prion protein gene (PRNP D178N), along with methionine at the 129 codon polymorphism on the mutant allele [46]. People with the familial natural short sleepers phenotype rise very early in the morning but maintain a bedtime similar to that of normal sleepers, leading to a reduction of 2 h in their daily sleep. These people possess mutations in DEC2, a gene involved in circadian rhythms by inhibiting CLOCK/BMAL activity and repressing hypothalamic secretin (Hcrt) transcription [47].

However, sleep is a multifaceted behavior affected by genetic and environmental influences. While many sleep disorders have a genetic basis, they are often the result of interactions between genetic, environmental, and biological factors. Polymorphisms in circadian rhythms are present in some sleep disorders. Exploring the genetic aspects of these disorders could lead to insights into the mechanisms of sleep.

### 3.3 Sleep Disorders and Sleep–Wake Dysregulation

Decades of research have implicated that there is the alteration of sleep–wake regulation in insomnia state. Sleep–wake balance plays a crucial role in maintaining normal sleep and contributes to sleep pathophysiology [48, 49]. New findings are starting to reveal the complexities of sleep–wake dysregulation in sleep disorders, highlighting their crucial roles in insomnia. Furthermore, the sleep–wake system has been extensively researched for its vital functions in sleep health and diseases [50].

A lot of nodes in sleep/wake regulation play important role in sleep disorders. The VTA‐dopaminergic neuron activation is acknowledged as sufficient to promote wakefulness and necessary to maintain it [51, 52]. Existing research indicates that in the current social context, many individuals face sleep–wake disorders due to sleep deprivation [53]. For instance, “forced sleep deprivation” such as night shifts, jet lag, or “nonforced sleep deprivation” caused by nighttime light sources (e.g., television, computers, and smartphones) can disrupt the normal sleep cycle, leading to the development of sleep–wake disorders.

### 3.4 Sleep Disorders and Impaired Glymphatic System

The glymphatic system, as a pathway reliant on glial cells for clearing metabolic waste, is well known to be mostly active during sleep [54]. Numerous recent studies have revealed a link between the glymphatic dysfunction and sleep disorders [55, 56]. Sleep deprivation decreases clearance of the removal of protein waste products including potentially toxic metabolites out of the brain through the glymphatic system [57, 58], indicating a compromised glymphatic system in the brain following sleep deprivation. A significant amount of evidence connects sleep disorders with pathophysiological processes that modify glymphatic clearance [59, 60, 61], which leads to which leads to toxic waste buildup, higher beta‐amyloid levels and a dysfunction in cognitive performance.

### 3.5 Molecular Mechanisms

#### 3.5.1 Neurotransmitter Changes Involved in Sleep Dysfunction

Brain imaging studies showed that sleep dysfunction involved in neurotransmitter systems of cerebral neural circuits. Good sleep is beneficial for the normal release of specific neurotransmitters, whereas sleep dysfunction affects the ability and sensitivity of the receptors [62, 63]. Sleep disturbance involves a complex neurotransmitter network (called the “sleep matrix”), which shares overlapping brain regions.

##### 3.5.1.1 Cholinergic Changes

Sleep dysfunction related to cholinergic alterations overlaps with neural circuits [64]. In individuals with sleep disorders, diverse changes in the cholinergic system can happen in various brain areas. It was reported that peoples with isolated REM sleep behavior disorder had hypercholinergic activity in mesopontine cholinergic neurons [64]. Bedard et al. [65] assessed cholinergic neuronal integrity in iRBD using PET （positron emission tomography） neuroimaging with the ^18^F‐fluoroethoxybenzovesamicol (FEOBV) and showed the brain cholinergic alterations in iRBD patients. Research has shown that there is an increase in cholinergic innervation in various regions in iRBD, with the most notable changes occurring in brainstem areas that promote REM sleep.

##### 3.5.1.2 Noradrenergic Changes

Brain noradrenergic circuit changes serve a significant function in the sleep behavior disorder. Noradrenaline level alterations in the brain play a significant role in brain dysfunctions and neurodegenerative disorders associated with REM sleep deprivation [66, 67, 68, 69]. The primary source of noradrenaline is from LC neurons. It is now well appreciated that there is a close correspondence between noradrenaline and REM sleep‐loss‐associated symptoms in patients.

The study by Andersen et al. examined if there was a relationship between the binding of the norepinephrine transporter ligand ^11^C‐MeNER and the loss of dopamine storage capacity in the nigrostriatal pathway, assessed using 18F‐DOPA PET, and found altered noradrenergic neurotransmission in the primary sensorimotor cortex (M1S1) of iRBD patients [70].

##### 3.5.1.3 Dopaminergic Changes

In patients, sleep disruptions can initiate manic episodes, and dopamine is a key factor in this transition [71], and sleep deprivation decreases binding of [^11^C]raclopride to dopamine D2/D3 receptors in the human brain [72]. Wu et al.’s findings indicate that dopaminergic pathways throughout the brain regulate changes in affective states caused by sleep deprivation, implying that dopamine pathways play a role in these transitions [73]. Using in vivo molecular imaging study, Stokholm et al. [74] investigated if extrastriatal monoaminergic systems are affected in iRBD patients and found that thalamic monoaminergic dysfunction in individuals with iRBD could signify terminal dysfunction in neurons projecting from the LC and dorsal raphe nucleus (DRN), which are responsible for REM sleep regulation.

#### 3.5.2 Neural Circuits Involved in Sleep Dysfunction

**FIGURE 2:**
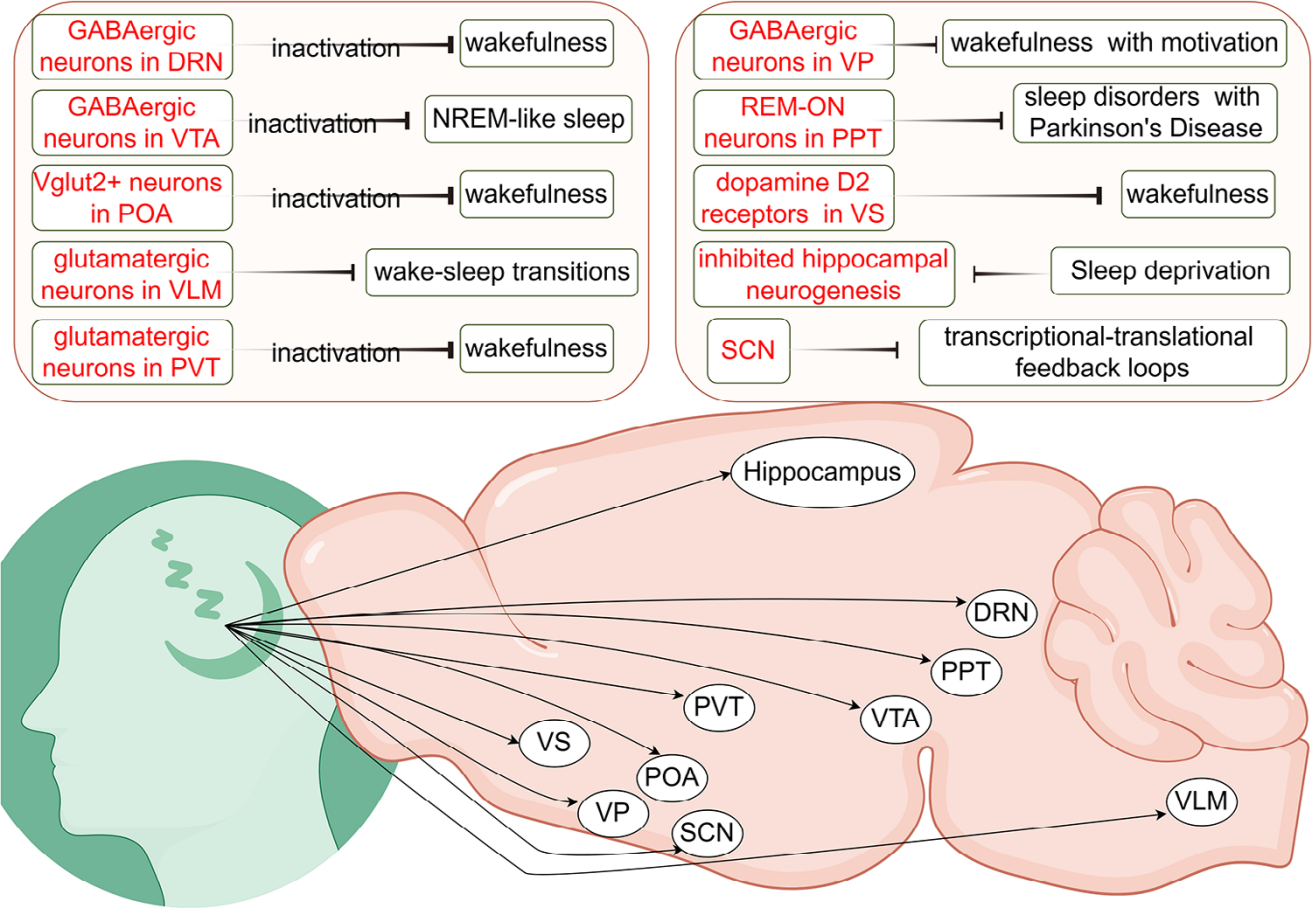
The brain subregions and nuclei implicated in sleep dysfunction. There are different neuronal types in DRN, VTA, POA, and other brain regions or nuclear clusters, which play different roles in sleep and wakefulness. DRN, dorsal raphe nucleus; POA, preoptic area; PPT, pedunculopontine tegmentum; PVT, paraventricular nucleus; SCN, suprachiasmatic nuclei; VLM, ventrolateral medulla; VP, ventral pallidum; VS, ventral striatum; VTA, ventral tegmental area.

The structure of brain appears in topographically organized groups. The expression of neurotransmitters is characterized by brain subdivisions, which are comprised cellular morphology, afferent and efferent projections, and functional properties. Here, we discuss the brain subregions (Figure 2) and related neural circuits implicated in sleep dysfunction (Table 2).

**TABLE 2:** A list of preclinical studies showing neuronal morphologic and functional changes in the sleep–wake states.

**Brain region**	**Neuron**	**Neural morphologic and functional changes**	**References**
Dorsal striatum	Dopamine D1R neurons	Striatal neurons expressing dopamine D1 receptor promote wakefulness	[115]
Nucleus accumbens	Dopamine D1R neurons	Neurons expressing dopamine D1 receptors in the nucleus accumbens are responsible for regulating wakefulness	[116]
Ventral pallidal	GABAergic neurons	Ventral pallidal GABAergic neurons control sleep–wake behaviors in mice	[102]
Ventral pallidum	CaMKIIa neurons	Stimulating CaMKIIa‐expressing neurons in the ventral pallidum enhances wakefulness	[117]
Ventral pallidal	Glutamatergic neurons	Ventral pallidal glutamatergic neurons regulate wakefulness	[118]
Preoptic hypothalamus	Glutamatergic neurons	Neurons that release glutamate in the preoptic hypothalamus encourage wakefulness	[85]
Ventrolateral medulla	Glutamatergic neurons	Nonrapid eye movement (NREM) sleep is regulated by glutamatergic neurons in the ventrolateral medulla that connect to the preoptic area	[86]
Ventral tegmental area	Dopaminergic neuron	Ventral tegmental area (VTA)‐dopaminergic neuron activation drives and maintains wakefulness	[52]
Ventral tegmental area	GABAergic neurons	The activity of VTA‐GABAergic neurons is closely associated with cortical arousal	[51]

##### 3.5.2.1 DRN‑Related Circuits

Through fiber photometry and concurrent electroencephalography/electromyography (EEG/EMG) recording, Cai et al. revealed that GABAergic neurons in the DRN displayed significant activity during wakefulness and reduced activity during nonrapid eye movement (NREM) sleep, indicating that GABAergic neurons, the second most prevalent cell type in the DRN, are involved in regulating sleep and wakefulness [75]. It is known that optogenetic stimulation or chemogenetic activation for DRN GABAergic neurons inducts and maintains wakefulness through the GABAergic DRN–VTA pathway. Circuit‐specific investigations from Ren et al. [76] demonstrated that GAD2‐positive GABAergic neurons in DRN constrained wakefulness by inhibiting the wakefulness‐promoting paraventricular thalamus (PVT), suggesting a wakefulness‐constraining role of DRN–PVT pathway.

##### 3.5.2.2 VTA‑Related Circuits

The VTA is a key region of the mesolimbic dopamine system involved in motivation, learning and reward processing [71, 73, 77]. VTA contains several neuron types, such as dopaminergic, glutamatergic/nitrergic (NOS1), and GABAergic neurons. It is demonstrated that VTA serves as an important node in sleep/wake regulation [78, 79]. Optogenetic activation of dopaminergic neurons in the VTA triggered and sustained wakefulness while inhibiting sleep and behaviors associated with sleep nesting [52].

Novel VTA‑related circuits have been screened in the mouse brain to promote wakefulness. Studies showed that there exist glutamatergic/nitrergic (NOS1) and GABAergic neurons in VTA by using chemogenetic activation experiments and electroencephalogram recordings, and these neurons are wake‐ and REM sleep‐active [80]. The stimulation of VTA glutamatergic/NOS1 neurons can lead to wakefulness via their connections to the NAs and lateral hypothalamus (LH). Further, activating GABAergic neurons in VTA elicited long‐lasting NREM‐like sleep resembling sedation.

##### 3.5.2.3 POA‑Related Circuits

The preoptic area (POA) is recognized as a key region that promotes sleep in mice [81, 82]. POA contains several neuron types, such as glutamatergic, GABAergic, and tachykinin 1 (Tac1) neurons [83, 84]. The inactivation of preoptic glutamatergic (Vglut2+) neurons initiates wakefulness, decreases NREM sleep, and causes a persistent suppression of REM sleep, whereas the activation of above neurons promotes arousals from sleep, suggesting that the POA plays a causal role in controlling both sleep and wakefulness [85]. The study by Teng et al. found that chemogenetically silencing POA glutamatergic‐positive neurons leads to a reduction in NREM sleep, while activating them promotes it [86].

There is unequivocal agreement that the lateral preoptic area (LPO) a center for NREM sleep homeostasis [87]. Miracca et al. [88] showed that NMDA receptors in the LPO are essential for sustaining NREM and REM sleep, and deleting the NMDA receptor GluN1 subunit from LPO abolished calcium signals to produce insomnia. Further, neurons that release GABA in the ventrolateral POA are crucial for starting and sustaining sleep, and they form direct synaptic links with arousal neurons (including orexin‐producing neurons and histaminergic neurons) in the lateral posterior hypothalamus [89, 90, 91].

##### 3.5.2.4 VLM‑Related Circuits

Many studies have shown the ventrolateral medulla (VLM)‑related neural circuits involved in sleep dysfunction. In addition to glutamatergic cells, neurons containing GABA, dopamine, tryptophan hydroxylase (TPH), and TH have been characterized in the VLM [51, 52, 92–94].

Teng et al. [86] identified a population of sleep‐active glutamatergic neurons in the VLM that project to GABAergic neurons in the POA, and their research revealed a stimulating circuit between the brainstem and hypothalamus that regulates transitions between wakefulness and sleep.

##### 3.5.2.5 PVT‑Related Circuits

The PVT of the thalamic midline is recognized as a key component in networks that regulate circadian rhythms and sleep/wake cycles. PVT contains several neuron types, such as glutamatergic, cholinergic, and oxytocin receptor‐expressing neurons [95, 96, 97].

Neuroscience is especially interested in the particular nucleus and neural pathways that regulate wakefulness [98, 99, 100]. Ren et al. [101] showed that PVT glutamatergic neurons exhibited high activities during wakefulness, whereas suppression of PVT neuronal activity caused a reduction in wakefulness. Further, activating glutamatergic neurons in PVT induced a transition from sleep to wakefulness, and activation of PVT neurons accelerated the revival from general anesthesia. The activation of PVT glutamatergic neurons can control wakefulness through projections to the NAs (PVT–NA pathway) and hypocretin neurons in the LH (PVT–LH pathway).

##### 3.5.2.6 VP‑Related Circuits

Sleep dysfunction is often comorbid with depression, and their underlying neuronal circuit mechanism have generated much interest. Ventral pallidum (VP) contains some neuron types, such as GABAergic, CaMKIIa, glutamatergic neurons. Li et al. reported that GABAergic VP neurons control wakefulness associated with motivation [102].

##### 3.5.2.7 PPT‑Related Circuits

Clinical observation showed that sleep disorders in patients with PD often accompanied by abnormal function of the pedunculopontine tegmentum (PPT) [103, 104].

It is known that the PPT is the site of REM‐ON neurons [105]. PPT contains several neuron types including dopaminergic, glutamatergic, cholinergic, and melanocortinergic neurons [106, 107, 108]. Separate studies have implicated the PPTg in processing aversive stimuli to dopamine systems.

##### 3.5.2.8 Ventral Striatum‑Related Circuits

Evidence that sleep deprivation downregulates dopamine D2R in VS in the human brain [109]. It is well known that dopamine D2 receptors in VS are involved with wakefulness. The study by Guo et al. revealed a significant reduction in the functional connectivity (FC) between the NAs (a part of the VS) and the ventrolateral periaqueductal gray (vLPAG) 6 h postsleep deprivation, suggesting that VS–vLPAG pathway may play an important role for sleep loss [110].

##### 3.5.2.9 Hippocampus‑Related Circuits

It is well acknowledged that prolonged sleep deprivation has detrimental effects for the hippocampus [111, 112, 113]. Growing evidence suggests that the hippocampus is divided both anatomically and functionally along a dorsolateral axis for cognitive functions and a ventromedial axis for mood and stress response control [114]. Employing the “platform on the water” method, extended sleep deprivation leads to a decrease in hippocampal cell growth and neurogenesis in both the dorsal and ventral regions of the dentate gyrus (DG) [111]. Two weeks of sleep deprivation results in suppressed neurogenesis in the hippocampus and a decrease in dendritic spine density in the DG [25].

#### 3.5.3 Cellular Mechanisms Involved in Sleep Dysfunction

Cellular changes caused by sleep dysfunction are the basis for functional and structural changes in brain regions and circuits. Changes in neuronal plasticity and glial activation in brain structures play a role in sleep disorder.

##### 3.5.3.1 Neurons

The use of ^13^C‐nuclear magnetic resonance (NMR) spectroscopy in combination with the intravenous infusion of [1‐^13^C] glucose, predominantly consumed by neurons [119, 120], has emerged as a significant method to explore neuronal potential metabolism in mice associated with sleep dysfunction. Using [1‐^13^C] glucose, the metabolic kinetics analysis from Zhu et al. [22] showed that chronic sleep deprivation (CSD) led to a reduction in neuronal Glu4 and GABA2 synthesis, which could be entirely restored through the forced expression of Sirt6. Using whole‐cell patch clamp recordings, Zhu et al. [22] examined miniature excitatory postsynaptic currents (mEPSCs) and action potential (AP) firing rates, finding that CSD caused a decrease in both AP firing rates and the frequency and amplitude of mEPS in the prelimbic cortex (PrL) pyramidal neurons, which could be partly reversed by Sirt6 overexpression.

Representative synaptic plasticity involves changes in synaptic strength that depend on activity, including long‐term depression (LTD), long‐term potentiation (LTP), and the formation of neocortical dendritic spines and synapses [121, 122, 123]. The research by Gao et al. revealed that chronic sleep restriction (CSR) in mice led to impaired synaptic plasticity, marked by lower synaptophysin and PSD95 levels and thinner postsynaptic density [124].

In patients, disruptions of sleep can elicit rapid manic onset, hyperactivity, and elevated social behaviors [73]. Using a hybrid automated sleep deprivation platform, Wu et al. [73] observed the modification of synaptic plasticity in the medial prefrontal cortex after acute sleep loss, and showed that disruptions of sleep‐induced dopamine‐dependent enhancement in dendritic spine density whereas uncaging of sleep deprivation evoked dendritic spinogenesis. Using sparse labeling of adult neurons to determine the density of dendritic spines in the DG, Jia et al. [25] found that SIRT6 improves hippocampal neurogenesis following prolonged sleep deprivation through modulating energy metabolism in developing rats.

##### 3.5.3.2 Microglia

There is considerable evidence that noradrenaline (NA) plays a vital role in sleep dysfunction. NA is well known a key modulator of microglia [66], and brain imaging studies showed that sleep disruption‐induced elevated noradrenaline play some roles in the initiation and development of neurodegeneration from molecule to pathophysiological changes [125]. Stokholm et al. [74] observed significantly raised microglial activation in the occipital lobe of iRBD patients.

Ma et al. [126] studied sleep interaction reciprocally with immune system activity, and found in mice that microglia, the resident immune cells in the brain, regulated sleep through a mechanism involving Gi‐coupled GPCRs, intracellular Ca^2+^ signaling and suppression of norepinephrine transmission. Liu et al. [127] found that propofol reduced sleep deprivation‐induced cognitive impairment and circadian rhythm disruption, possibly by lowering neuronal inflammation and switching the microglia phenotype from an M1 to an M2 activated state, thus exerting neuroprotective effects.

##### 3.5.3.3 Astrocyte

CSD‐induced activated astrocyte plays a significant role in neuronal plasticity and cognitive behaviors. Recent studies have focused on the ^13^C‐NMR spectroscopy analysis of cerebral metabolism in many diseases [128, 129, 130, 131]. Metabolic kinetics studies showed that CSD engages the change of astrocyte activity. Because [2‐^13^C]‐acetate is taken up, utilized and metabolized exclusively by astrocytes [132, 133, 134], a metabolic kinetics analysis based on NMR spectroscopy is used to study astrocyte metabolism after CSD. Zhu et al. [22] found that Sirtuins 6 (Sirt6) improved cognitive impairment after CSD by regulating the astrocytic glutamatergic neurotransmission.

## 4 Sleep Disorders and Celiac Disease

CD is an immune‐mediated enteropathy induced by gluten consumption in individuals genetically predisposed with human leukocyte antigens (HLAs) DQ2 or DQ8. Increasing evidence indicates a strong association between gut microbiota and gastrointestinal disorders, such as CD. Alterations in the gut microbiota of HLA‐DQ2/8 individuals can impact gluten processing in the lumen, influencing the intestinal barrier and immune responses, potentially triggering or worsening gluten‐sensitive enteropathy [135]. Flora imbalance is an imbalance between protective and pathogenic microorganisms in the host. Initially, small bowel biopsies from patients with both active and inactive CD showed an increase in rod‐shaped bacteria.

Fecal cultures and duodenal biopsies revealed elevated levels of Gram‐negative, anaplastic, clostridial, and *Escherichia coli* bacteria in CeD patients compared to healthy adults. The Swedish CeD Epidemiologic Study reinforced the notion that dysbiosis is a risk factor for CeD, identifying elevated levels of rod‐shaped bacteria such as *Clostridium* spp., *Prevotella* spp., and *Actinobacteria* spp.in the small intestinal mucosa of CD patients [135]. Individuals with CD frequently experience severe and debilitating fatigue as their primary symptom. Fatigue is often described as a state of prolonged exhaustion accompanied by an inability to perform activities at the expected capacity, resulting in sleep deprivation or sleep disorders; depression or anxiety; and impaired cognition, motivation, attention, and physical abilities. Neuropsychiatric disorders, including schizophrenia, depression, and anxiety, have been linked to CD. Patients with CD exhibit a cortical arousal pattern marked by “disinhibition” and “hyperfacilitation.” Dysregulation of the immune system may play a central role in triggering changes in motor cortex excitability. In CD patients, antimyelin antibodies may disrupt the balance of excitatory and inhibitory neural circuits by interacting with synaptic protein I. TMS, alongside clinical cognitive, immunological, and imaging data, serves as an additional tool to detect subtle changes in the pathophysiological and neurochemical mechanisms of excitatory gut–brain connectivity [136].

## 5 Sleep Disorders and Hepatic Disease

As the most important biochemical reaction and metabolic site in the human body, the liver also undergoes diurnal rhythmic changes under physiological conditions [137]. Unlike other organs that are primarily regulated by the “master clock” in the SCN, the liver possesses an endogenous circadian rhythm known as the “liver clock,” enabling the liver to maintain diurnal rhythms in gene expression, protein synthesis, and other aspects for a considerable period even when deprived of SCN regulation [138, 139]. In this context, the close relationship between the occurrence and development of liver diseases and the circadian rhythm of organisms mutually promoting and causative factors is not surprising. Existing research indicates that in the current social context, many individuals face sleep–wake disorders due to sleep deprivation [53]. For instance, “forced sleep deprivation” such as night shifts, jet lag, or “nonforced sleep deprivation” caused by nighttime light sources (e.g., television, computers, and smartphones) can disrupt the normal sleep cycle, leading to the development of sleep–wake disorders. Epidemiological data have confirmed that over half of cirrhotic patients suffer from sleep–wake disorders, with the severity worsening as the degree of cirrhosis progresses [140]. This sleep–wake disorder can promote the occurrence of liver diseases, especially for patients already suffering from chronic liver insufficiency or cirrhosis [140, 141]. A meta‐analysis of six studies showed an association between sleep duration and nonalcoholic fatty liver disease (NAFLD) [142]. Similarly, chronic liver insufficiency or cirrhosis can reciprocally induce or exacerbate sleep–wake disorders [142, 143, 144]. Endogenous melatonin produced by the pineal gland is the main mediator of the neural–humoral regulation of circadian rhythms in organisms [144]. During daylight conditions, multiple neural pathways transmit perceived information to the pineal gland via the SCN, inhibiting melatonin production. As night falls and light diminishes, this inhibition is relieved, allowing for melatonin synthesis and release [145]. In humans, the rhythmic variation of melatonin is one of the most direct evidence of circadian rhythms in the body. Synchronization with the biological clock enables melatonin to adapt to the physiological activities of the body during different periods to meet physiological needs. For example, melatonin can increase sleep tendency by influencing SCN neuronal activity. Under normal physiological conditions, the rise and fall of melatonin levels in the body are consistent with the intensity of light. However, compared to the normal control group, cirrhotic patients exhibit delayed production of endogenous melatonin, characterized by later secretion timing and peak levels, indicating a lag in peak time [146]. Obviously, the phenomenon cannot be solely explained by liver dysfunction itself but rather by the presence of circadian rhythm disorders in the liver or a disruption of the SCN rhythm. Not only the abnormal changes in the rhythmic secretion of melatonin but also the disturbance of internal environmental homeostasis, RLS, and abnormal temperature regulation caused by liver dysfunction can contribute to the occurrence of sleep–wake disorders.

**FIGURE 3:**
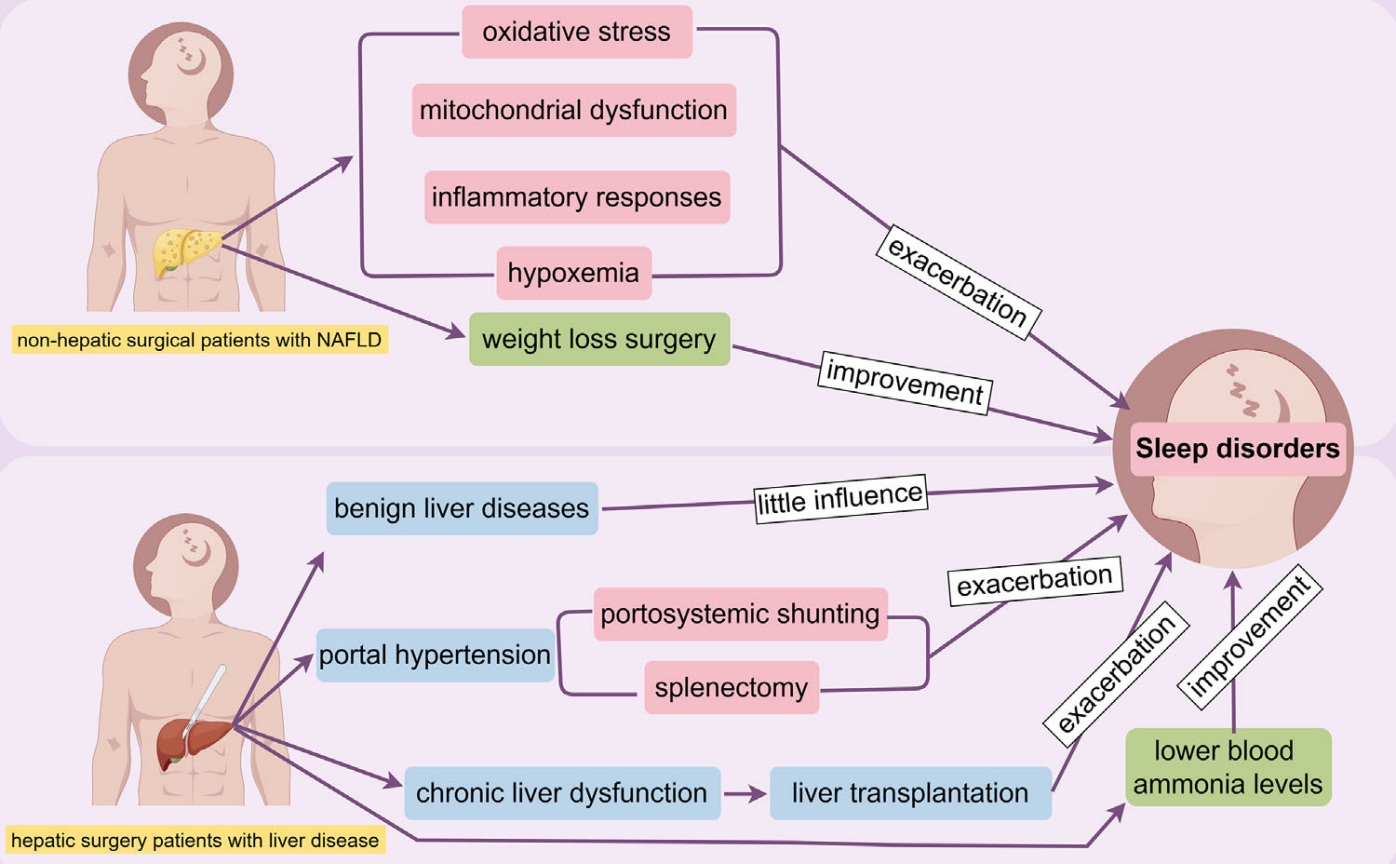
Perioperative sleep–wake disorders in nonhepatic and hepatic surgical patients with liver disease. Due to the direct or indirect impact of the disease itself, most of these patients already suffer from varying degrees of sleep–wake disorders before surgery. After undergoing surgery and anesthesia, they may face even worse sleep–wake disorders postoperatively. But a small percentage of people with sleep disorders may benefit from liver surgery. NAFLD, nonalcoholic fatty liver disease.

The combination of chronic liver disease patients in clinical anesthesia is not uncommon. Due to the direct or indirect impact of the disease itself, most of these patients already suffer from varying degrees of sleep–wake disorders before surgery. After undergoing surgery and anesthesia, they may face even worse sleep–wake disorders postoperatively. This review will provide a comprehensive discussion of perioperative sleep–wake disorders in patients with chronic liver insufficiency (Figure 3).

### 5.1 Perioperative Sleep–Wake Disorders in Nonhepatic Surgical Patients With Liver Disease

Research on perioperative sleep–wake disorders in patients with chronic liver dysfunction undergoing nonhepatic surgery is mainly focused on NAFLD patients. This section will summarize the progress of such research.

NAFLD is characterized by hepatic steatosis without significant alcohol consumption history, excluding syndromes caused by alcoholic liver disease [147, 148, 149]. NAFLD, a prevalent liver disease worldwide, is strongly linked to obesity, type 2 diabetes, hyperlipidemia, sleep apnea, and a sedentary lifestyle [150, 151, 152]. If not controlled in time, NAFLD can progress to cirrhosis or even hepatocellular carcinoma [153]. Sleep apnea and hypopnea syndrome are often coexisting conditions with NAFLD [154, 155, 156]. Patients with these conditions exhibit abnormal respiratory rhythms and ventilation during sleep, leading to nocturnal awakenings and significantly reduced sleep quality. Consequently, most patients experience symptoms of sleep–wake disorders such as daytime sleepiness, drowsiness, and cognitive impairment [157, 158, 159, 160]. This may be closely related to oxidative stress, mitochondrial dysfunction, and activation of inflammatory responses caused by nocturnal hypoxemia, which in turn lead to insulin resistance, metabolic abnormalities in the liver, and atherosclerosis [161, 162, 163].

Currently, the treatment methods for patients with these comorbidities mainly focus on controlling the underlying causes and improving lifestyle factors such as weight loss, diabetes control, and lipid control. Studies have shown that even modest weight loss can improve hepatic steatosis, liver metabolism, and sleep apnea [164, 165, 166]. Weight loss surgeries such as gastric bypass and sleeve gastrectomy have been proven to alleviate or even reverse hepatic steatosis in NAFLD patients while improving metabolic syndrome [167, 168]. Additionally, weight loss surgery can improve sleep quality and sleep patterns by reducing the frequency of nocturnal apneas and increasing tidal volume, thus alleviating sleep–wake disorders in obese patients with fatty liver disease. Therefore, some scholars recommend weight loss surgery for patients with a BMI (Body Mass Index) greater than 35 to improve their liver condition [169, 170, 171].

### 5.2 Perioperative Sleep–Wake Disorders in Hepatic Surgery Patients With Liver Disease

Patients with benign liver diseases such as hepatic hemangiomas generally experience no significant changes in perioperative sleep conditions compared to those undergoing other surgeries. The main reasons for this are typically attributed to preoperative anxiety and postoperative pain. In contrast, patients with portal hypertension face more severe sleep–wake disorders during the perioperative period. This section will discuss this in detail.

Portal hypertension can result from various conditions, including liver cirrhosis and schistosomiasis, which lead to obstruction or increased blood flow in the portal vein. Portal hypertension is diagnosed when the portal vein pressure exceeds 25 cm H_2_O [173, 174]. Splenomegaly, hypersplenism, esophageal varices, hormonal disturbances, and ascites are common complications of portal hypertension [175, 176]. Besides controlling the underlying liver disease and lifestyle interventions, surgical treatment is an essential component of managing portal hypertension. This includes procedures to alleviate portosystemic shunting, such as transjugular intrahepatic portosystemic shunt (TIPS) and nonselective portocaval shunt, as well as splenectomy for splenomegaly and hypersplenism [177, 178, 179]. However, surgery may exacerbate or accelerate the development of hepatic encephalopathy in patients with chronic liver dysfunction. Studies have shown that patients with extensive portosystemic shunting or those who have undergone portosystemic shunt procedures to relieve portal hypertension are more prone to developing hepatic encephalopathy [178, 180, 181]. This is because blood from the intestines bypasses the liver through the portosystemic shunt, leading to increased blood ammonia levels, which promotes neurological damage and facilitates the progression to hepatic encephalopathy. Consequently, these patients are also more likely to experience sleep disturbances [182, 183, 184]. Although patients with chronic liver dysfunction and cirrhosis also experience sleep–wake disorders, the close association between hepatic encephalopathy and sleep–wake disorders suggests a more intimate relationship between the two, possibly sharing some common pathological and physiological mechanisms. The primary hypothesis for the pathogenesis of hepatic encephalopathy is the ammonia theory. In a clinical study, patients with cirrhosis were administered a loading dose of amino acids to induce elevated blood ammonia levels, and their sleep was monitored and recorded using methods such as electroencephalography. The results showed that compared to the normal control group, patients with cirrhosis exhibited significant sleep–wake disorders, including reduced total sleep time and increased frequency of awakenings during sleep [185].

In conclusion, patients with portal hypertension are more likely to experience sleep disturbances after surgical treatment in the terminal stage, which is closely related to their increased susceptibility to hepatic encephalopathy.

### 5.3 Perioperative Sleep–Wake Disorders in Liver Transplant Patients

Perioperative sleep–wake disorders in liver transplant patients have been a topic of growing interest in recent years (Table 3). Since the first liver transplant was performed in the United States in the 1960s, liver transplantation in China began in the 1990s. As an important treatment for end‐stage liver disease, primary liver cancer, and fulminant hepatitis, liver transplantation has become increasingly mature. Along with the maturation of liver transplantation and the widespread adoption of perioperative medical care and accelerated recovery surgical concepts, researchers have expanded their focus to include the perioperative period of liver transplantation, emphasizing the postoperative quality of life of patients. A meta‐analysis covering 65 clinical studies showed that the incidence of sleep–wake disorders after liver transplantation in patients with chronic liver dysfunction could be as high as 28% [186]. Sleep–wake disorders may cause fatigue and decrease daytime attention and physical strength in liver transplant recipients. However, this disease has not received sufficient attention in clinical practice, with low diagnosis rates and difficulty in effectively managing diagnosed patients [187]. Patients undergoing liver transplantation need lifelong immunosuppressive therapy. Classic antirejection drugs commonly used after liver transplantation, such as mycophenolate mofetil, can cause insomnia in patients [188]. Calcineurin inhibitors and rapamycin, represented by cyclosporine and tacrolimus, can cause symptoms such as headache, nausea, and vomiting, which can severely affect the quality of sleep in patients and induce sleep–wake disorders [189]. Studies have shown that patients with concomitant psychiatric disorders are at higher risk of developing sleep–wake disorders after organ transplantation than those without psychiatric history [190]. Similarly, these antirejection drugs can also cause patients to develop depression, anxiety, and other mental symptoms, possibly in a mutually causal relationship. In addition, sleep–wake disorders may lead to a decrease in medication adherence in patients, resulting in poor prognosis [191].

**TABLE 3:** Sleep–wake disturbances after liver transplantation.

	**Author**	**Year**	**Key findings**	**References**
1	Li et al.	2012	Patients who spent more than 3 years on the organ transplant waiting list were more likely to show tendencies of sleep–wake disorders	[210]
2	Fredericks et al.	2012	Sleep problems were common in pediatric liver transplant recipients and predicted significant variance in health‐related quality of life	[211]
3	Akahoshi et al.	2014	64% recipients were suffered from sleep disorder after liver transplantation	[212]
4	He et al.	2015	Sleep problems (41.2%) were common among children recipients after living donor liver transplantation	[213]
5	Ahmed et al.	2022	Anxiety and sleep disturbance continuously for 12 months after liver transplantation	[214]
6	Lim et al.	2023	The factors associated with the poor sleep quality of the liver transplant recipients included depression and symptom experience	[191]

Hepatic encephalopathy, mainly secondary to cirrhosis, is a severe complication of various end‐stage liver diseases [192]. Its early clinical manifestations mainly include changes in personality, cognition, and motor function. The most commonly used clinical grading of hepatic encephalopathy is the West Haven criteria (WHC), which comprehensively differentiate symptom changes in patients with different degrees of hepatic encephalopathy, including personality changes, mental abnormalities, behavioral abnormalities, speech abnormalities, consciousness status, electroencephalogram changes, and typical symptoms (such as flapping tremor) [193]. As the disease progresses, further deterioration of liver function will lead to intracranial tissue edema. At this stage, advanced cortical function is severely impaired, and patients may experience consciousness disorders or even enter a coma [194, 195]. The “ammonia toxicity” hypothesis is one of the important pathogenic mechanisms of hepatic encephalopathy recognized by the academic community. Elevated levels of blood ammonia and various inflammatory factors may increase blood–brain barrier permeability by promoting the breakdown of tight junctions within the blood–brain barrier [196, 197, 198]. Based on this, a large amount of ammonia enters the central nervous system and may induce (or exacerbate) brain tissue edema through various possible pathways, leading to astrocyte swelling, energy metabolism disorders, and triggering neuroinflammation. In addition to the main description of ammonia molecules, other toxic substances such as free tryptophan and pseudoneurotransmitters (β‐hydroxyphenylalanine and phenylethanolamine) are also involved in the occurrence and development of hepatic encephalopathy [199, 200]. Generally, most hepatic encephalopathy related to chronic liver dysfunction can be partially reversed after removing or alleviating precipitating factors. However, hepatic encephalopathy caused by fulminant liver failure is often difficult to control or reverse due to a rapid increase in blood ammonia levels, leading to diffuse brain edema and brainstem organic damage, which are difficult to reverse [201, 202].

Patients undergoing liver transplantation usually have end‐stage liver disease, severe liver dysfunction, cirrhosis, or hepatic encephalopathy. These complications can also cause patients to develop sleep–wake disorders. In the 1950s, Sherlock et al. first reported sleep–wake disorders in patients with hepatic encephalopathy [203]. According to multiple studies on the sleep status of patients with hepatic encephalopathy, approximately 50%–80% of patients with hepatic encephalopathy have sleep disorders [204, 205, 206].

Clinical studies have shown that treatments to lower blood ammonia levels, such as oral lactulose and rectal acidic solution enemas, can improve sleep quality and structure [207, 208]. Elevated blood ammonia levels have also been shown to be associated with slowing of electroencephalographic activity during wakefulness, exhibiting a pattern similar to the transition to sleep. Currently, the main clinical manifestations of sleep–wake disorders related to hepatic encephalopathy include sleep–wake reversal, daytime sleepiness, difficulty falling asleep at night, and easy awakening [209].

In addition to objective pathological and pharmacological factors, the mental and psychological status of patients during the perioperative period may also affect and lead to the occurrence of sleep–wake disorders. First, patients who need liver transplantation mostly have end‐stage liver disease, and their quality of life has been severely affected by the disease. Second, due to the shortage of liver donors, these patients need to wait for a long time on the organ transplantation list to obtain suitable liver sources. During this period, patients often face psychological pressure from disease, economics, and family. A clinical survey from Hong Kong, China, showed that the length of time patients spent on the organ transplant waiting list was closely related to the incidence of mental disorders. Patients who waited for more than 3 years were more likely to show tendencies of depression, anxiety, and insomnia [210]. This mental state can also affect the perioperative period of patients, especially the preoperative sleep status. Finally, patients who successfully match and receive liver transplants also face long‐term use of antirejection drugs, lifestyle restrictions, and the economic burden of long‐term diagnosis and treatment, making them more likely to experience somatization and emotional disorders during the perioperative period.

## 6 Therapeutic Interventions for Sleep Disorders

With the development of life‐fast pace, more and more patients suffer from sleep disorders. As the most common sleep disorders, the prevalence of insomnia has increased significantly in recent years, and the importance of emerging therapeutic approaches for sleep–wake disorders has gradually become prominent. Because of the increasingly detailed classification of medical disciplines, there are such problems as the low degree of discipline integration, resulting on the lack of effective treatment for intractable insomnia. Innovative pharmacological and neurostimulation strategies may focus on the cholinergic system to address sleep–wake disorders.

### 6.1 Pharmacological Interventions

Some drugs have shown a regulatory effect on insomnia to some extent by regulating the levels of neurotransmitters. Benzodiazepine drugs, such as diazepam, focus on the side effects of long‐term use based on GABA receptor activation. Nonbenzodiazepine drugs such as zolpidem and zaleplon also act on GABA receptors, but their effects are more short‐term, reducing the risk of addiction.

Excessive and/or inappropriately timed appetitive hormone release disrupts wakefulness–sleep states and muscle control during sleep. Overexcitation of appetitive hormone neurons is responsible for increased sleep fragmentation, although the number of appetitive hormone neurons decreases with age [215]. Preclinical evidence suggests that orexin is critical for maintaining the active wakefulness behaviors that drive sleep demand [216]. Orexin neurons are active during wakefulness, especially during exploration, but are mostly inactive during sleep, except for sporadic bursts during REM sleep, which may coincide with muscle twitches. In obstructive sleep apnea syndrome (OSAS), sleep fragmentation is primarily caused by recurrent interruptions. Recurrent intermittent hypoxia or hypercapnia from OSAS can activate orexin neurons, causing arousal and sleep fragmentation. In RLS, elevated levels of orexin‐A have been found in evening cerebrospinal fluid samples, particularly in early‐onset disorders, although follow‐up studies have not confirmed these findings and have not shown any correlation with symptom severity and polysomnographic measurements. Nonetheless, circadian rhythmic changes in orexin A may be dysregulated in early‐onset RLS, with elevated nocturnal levels [217]. The complex interconnections between cancer, episodic somnambulism, and neurodegenerative diseases have recently been proposed as possible cases of appetitive hormone‐dependent reverse comorbidity. The use of appetitive hormone A in RLS has been recognized as a potential cause of the disease.

In the last 10 years, understanding the anatomical links between the orexin system and sleep/wake as well as motivational and reward circuits has been crucial for developing orexin receptor antagonists to treat insomnia. The identification of these interconnections has facilitated the creation of a novel system for treating insomnia. The orexin system, as previously noted, emerges as a promising therapeutic target due to its influence on sleep, addiction mechanisms, stress, mood, energy balance, and arousal systems. The dual orexin receptor antagonists (DORA) and selective orexin receptor antagonists (SORA) polysomnography systems significantly influence sleep and wakefulness. Research on sleep architecture in patients using DORA and SORA indicates that DORA enhances total sleep time mainly by increasing REM sleep, while it either does not affect or reduces non‐REM sleep. Research indicates that DORA positively influences the sleep structure of patients treated with both DORA and SORA. However, the limited studies on SORA alone prevent definitive conclusions. With regard to its efficacy and safety, DORA has performed well, although systematic comparisons between these drugs are rare [218].

Taken together, the diverse biological roles of orexins imply that orexin receptor antagonists may influence various functions and potentially improve or exacerbate comorbidities. Insomnia frequently co‐occurs with other disorders and shows significant individual differences in clinical characteristics, sleep patterns, and associated conditions. Appetite receptor antagonists have been demonstrated to enhance polysomnography (PSG) parameters, somnolence, and sleep quality, contingent upon patient characteristics. Thus, these drugs provide an interesting perspective for a targeted and individualized approach.

### 6.2 Cognitive Behavioral Therapy and Behavioral Interventions

Cognitive behavioral therapy is a technique aimed at improving sleep quality by altering negative cognitive and behavioral patterns. Mechanisms of Cognitive Behavioral Therapy for Insomnia (CBT‐I) have showed to reduce sleep anxiety by educating patients about sleep knowledge, timing control, cognitive restructuring, and so forth, enabling patients to better regulate their sleep. As an alternative to medication, CBT‐I has been shown to have significant effects in long‐term improvement of insomnia, and the underlying side effects are much smaller than drug treatment.

Relaxation techniques and mindfulness meditation improve sleep quality by reducing anxiety and stress, enhancing physical and mental relaxation. Deep breathing, gradually relaxing muscles, and guiding thoughts, can reduce sympathetic nervous activity, and parasympathetic nervous activity is increased, promoting sleep. Multiple studies have shown that mindfulness meditation can effectively alleviate insomnia, significantly improve sleep quality, and reduce sleep latency.

The balance of body and emotions can alleviate anxiety and stress reactions in patients with insomnia through cognitive behavioral therapy, relaxation exercises, and regulate their psychological state to enhance their psychological resilience, and promote better sleep.

### 6.3 Emerging Therapeutic Approaches

The neuromodulation therapy is an emerging therapeutic approach for sleep disorders. Neuromodulation methods include brain stimulation and neurofeedback therapy. Brain stimulation therapy is a technique that regulates neural activity and optimizes sleep structure and quality by directly or indirectly stimulating specific areas of the brain.

TMS is a noninvasive brain stimulation technique that uses a strong magnetic field to stimulate the cerebral cortex and regulate neural activity [219]. TMS generates a magnetic field through electrical stimulation, which affects neuronal activity in the cerebral cortex. TMS at specific frequencies (high or low) can promote or inhibit activity in specific brain regions. For example, high‐frequency stimulation of the left prefrontal cortex is associated with improving symptoms of depression, anxiety, and corresponding sleep quality. The application of TMS in insomnia patients has shown good results. Some studies have shown that TMS can improve sleep quality, bedtime, and nighttime awakenings.

Transcranial electrical stimulation (tDCS) is a method of regulating the excitability of the cerebral cortex by applying a weak direct current. tDCS enhances or inhibits neural activity by altering the polarization state of neurons. For example, stimulating the prefrontal cortex of the brain can promote sleep. Clinical trials have shown that tDCS can significantly improve the total sleep time and sleep efficiency of patients with insomnia symptoms, and theoretically reduce the degree of insomnia by affecting relevant neural networks.

Neurofeedback therapy helps individuals to learn how to regulate their own brainwave patterns to improve insomnia by monitoring and providing real‐time feedback on their brainwave activity. Mechanisms of neurofeedback therapy involve the following aspects: measuring of brain activity through EEG and providing feedback to individuals, prompting them to adjust their brainwaves to achieve a relaxed and sleep promoting state. Some studies have shown that neurofeedback therapy is effective in improving sleep and alleviating insomnia symptoms, especially in anxiety and stress‐related insomnia.

### 6.4 Anesthesia Technology

Rios et al. [13] demonstrated that propofol can enhance SWS, marked by significant electroencephalographic oscillations, and alleviate late‐life depression in certain individuals. Smith et al. [220] found that daytime sedation with dexmedetomidine combined with closed‐loop acoustic stimulation affected SWS homeostasis in healthy adults, indicating that this approach induces sleep‐like states meeting specific homeostatic NREM sleep requirements in young adults. Rios et al. [221] proposed a phase I open‐label trial investigating the enhancement of SWS with propofol to address geriatric depression and cognitive dysfunction, supported by a recent small case series. Anesthesia‐induced sleep balance technology is a method of treating stubborn insomnia by using the anesthetic drug propofol to induce sleep balance recovery, followed by targeted physical and drug treatment to restore the patient's natural sleep cycle. It is an important supplement to existing insomnia treatment methods.

## 7 Conclusion and Prospects

The mechanism of therapeutic interventions for sleep disorders needs further research. Neuromodulation therapy mainly improves sleep quality by regulating the activity of specific neurons and neurotransmitters. For example, TMS and tDCS directly stimulate relevant brain regions to affect the release of GABA and other neurotransmitters, further enhancing inhibitory nerve conduction, thereby promoting sleep onset and improving sleep structure. Adjusting EEG activity patterns through techniques such as neurofeedback can monitor and regulate EEG waves in real‐time. By teaching patients to enhance theta and alpha waves (related to relaxation and sleep quality), sleep efficiency can be improved, anxiety and stress levels can be reduced, and sleep can be improved. Activating the biological clock regulation system TMS and other brain stimulation techniques in the brain can affect the activity of areas such as the hypothalamus, regulate the body's biological clock, adapt to healthy sleep rhythms, and improve daily performance.

There are still many unsolved mysteries in the sleep disorders, and future research directions can be expanded from the following aspects: Based on an individual's physiological data (such as genomics, psychological status, etc.) and biomarkers, we develop personalized neural regulation strategies to improve treatment efficacy. Investigating the combined application of different neural regulation methods, psychological therapy, and drug therapy, we explore their synergistic effects and achieve more comprehensive sleep disorders management. We need evaluate the long‐term safety and effectiveness of novel neuroregulatory therapies, especially their lasting impact on patients with chronic sleep disorders, in order to guide clinical practice. Through modern neuroimaging techniques such as fMRI, we aim to explore in depth how different neural regulation treatments affect brain regions and networks, in order to reveal the biological basis of sleep disorders.

The treatment methods for sleep disorders are rapidly developing, providing new hope for improving sleep disorders and enhancing quality of life. These methods not only improve sleep quality by directly regulating brain activity, but also affect individuals' psychological and physiological states through various mechanisms, demonstrating broad application prospects. With the deepening of research, it is expected to achieve more personalized and diverse sleep disorders treatment plans in the future, bringing good news to billions of patients with sleep disorders.

## Author Contributions

All the authors read and approved the final manuscript.

Concept and design: Yifan Jia and Hongbing Xiang.

Acquisition, analysis, or interpretation of data: Cheng Liu, Zhigang He, Yanqiong Wu, Yanbo Liu, and Zhixiao Li.

Drafting of the manuscript: Cheng Liu, Zhigang He, Yanqiong Wu, and Yanbo Liu.

Critical review of the manuscript for important intellectual content: Yanbo Liu, Zhixiao Li, Yifan Jia, and Hongbing Xiang.

## Ethics Statement

The authors have nothing to report.

## Conflicts of Interest

The authors declare no conflicts of interest.

## Data Availability

Data availability is not applicable to this review as no new data were created or analyzed in this study.
